# Balancing photosynthesis and photoprotection: The role of RppA in acclimation to light fluctuations in cyanobacteria

**DOI:** 10.1093/plphys/kiae553

**Published:** 2024-10-17

**Authors:** Pablo Ignacio Calzadilla, Fernando Unrein

**Affiliations:** Assistant Features Editor, Plant Physiology, American Society of Plant Biologists, Rockville, USA; Instituto de Fisiología Vegetal (INFIVE), Universidad Nacional de La Plata—CONICET, cc 327, 1900 La Plata, Buenos Aires, Argentina; Department of Earth and Environmental Sciences, Faculty of Science and Engineering, University of Manchester, M13 9PT Manchester, UK; Instituto Tecnológico de Chascomús (INTECH), CONICET-UNSAM, Chascomús CC 164 (B7130IWA), Buenos Aires, Argentina; Escuela de Bio y Nanotecnologías, Universidad Nacional de San Martín, 1650 San Martin, Buenos Aires, Argentina

In nature, photosynthetic organisms are exposed to a fluctuating environment where light continuously changes. These light changes can be triggered by the shading of sunlight by clouds or disturbances of water bodies caused by the wind. For instance, in turbid shallow lakes, only the upper water layer is illuminated, whereas most of the water column remains in the darkness. Under these conditions, photosynthetic planktonic microorganisms, like many cyanobacteria, spend most of their time in darkness and only occasionally have the opportunity to reach the illuminated surface. As a result, these organisms experience marked dark-light fluctuations that can affect photosynthetic performance.

Evolution has shaped different mechanisms in cyanobacteria to cope with light environmental changes. At the core of these acclimation processes is the coordinated regulation of photosynthetic pigment biosynthesis, such as chlorophylls (Chls) and carotenoids (CARs). Chls are fundamental molecules for photosynthesis due to their light-harvesting capacity. However, because of their photosensitivity, their excessive accumulation (or of their metabolic intermediates) can trigger reactive oxygen species (ROS) formation (Zhi et al. 2019). CARs can reduce ROS accumulation, participating in photoprotection (Zhu et al. 2010). Thus, balancing Chl and CARs levels (and the timely and precise control of their biosynthesis) can ensure cyanobacterial survival and growth in different ecological niches.

The biosynthesis of Chl involves different enzymatic reactions, the final step being catalyzed by the enzyme chlorophyll synthase (ChlG). The expression of *chlG* regulates the entire Chl metabolic pathway (Shalygo et al. 2009) and participates in PSII assembly (Chidgey et al. 2014; Knoppová et al. 2014). Consequently, ChlG is strongly linked to the biogenesis of the photosynthetic apparatus and photosynthetic activity. Understanding ChlG regulation could advance our knowledge of cyanobacteria ecological distribution and potentially improve their utilization as biotechnological platforms. However, although the regulation of Chl biosynthesis has been extensively studied in plants (Kobayashi and Masuda 2016), its control in cyanobacteria is still unclear. The search for novel transcriptional regulators for *chlG* is fundamental to understanding how these organisms acclimate to fluctuating environments.

In this issue of *Plant Physiology*, Yu et al. (2024) explored the transcriptional regulation of *chlG* in *Synechocystis* sp. PCC 6803 (hereafter *Synechocystis*) under dark-light fluctuations, identifying RppA as a key transcription factor in this process. First, the authors performed a yeast 1-hybrid library screening to identify proteins binding to the 143-bp DNA fragment upstream of the *chlG* starting codon. Two transcription factors were identified: RppA and cyAbrB1. RppA belongs to the OmpR response regulators subfamily, senses the redox state of the plastoquinone pool, and regulates the expression of photosynthetic genes (Li and Sherman 2000). Meanwhile, cyAbrB1 is essential for growth and cell survival, and, as such, fully segregated mutants of this gene are not viable (Ishii and Hihara 2008). Thus, Yu and collaborators focused their study on RppA, confirming its physical interaction with the *chlG* promoter by the electrophoretic mobility shift assay.

A *rppA* knockout (*rppA*-KO) mutant was generated in *Synechocystis* to investigate the role of RppA in vivo. The growth of *rppA*-KO was similar to the wild-type strain under continuous low light (25 *µ*mol photons m^−2^ s^−1^). However, under fluctuating low-light conditions (3 h dark: 1 h light), *rppA*-KO showed chlorosis and growth retardation. Chl biosynthesis impairments can induce oxidative stress and photodamage (Zhi et al. 2019). Thus, intracellular ROS contents were measured in both strains to address their growth differences. While ROS levels were the same between the *rppA*-KO and the wild type under continuous low light, higher levels of ROS were observed in the *rppA*-KO under fluctuating low-light conditions. Higher nonphotochemical quenching was also observed in the mutant strain under fluctuating light, suggesting a higher dissipation of energy in the photosynthetic apparatus when RppA is mutated. Exposure of the cell cultures to moderate light intensity (80 *µ*mol photons m^−2^ s^−1^) exacerbated the *rppA*-KO phenotype.

Yu and colleagues studied the effect of RppA on *chlG* expression during dark-light transitions by reverse transcription quantitative PCR. Light increased *chlG* transcriptional levels, but this induction was higher in the *rppA*-KO mutant compared with the wild type. Therefore, RppA represses *chlG* expression during dark-light transitions in *Synechocystis*. Chl precursors chlorophyllide and protochlorophyllide levels were also higher in the *rppA*-KO, confirming the role of RppA as a negative regulator of Chl biosynthesis. The increased accumulation of these metabolic intermediates could explain the oxidative stress observed in the *rppA*-KO. ROS levels can also be modulated by CARs content. Hence, the authors quantified CARs by HPLC analyses. No differences were observed in CAR contents between the *rppA*-KO and the wild-type strain under dark conditions or low light exposure. However, moderate light illumination increased CARs contents in the wild type but not in the mutant strain. Since CARs participate in photoprotection, their reduced contents in *rppA*-KO could also explain their higher ROS levels.

To address a possible role of RppA in regulating CAR metabolic pathway, the transcriptional levels of its biosynthetic genes were evaluated during dark-light transitions. The light-induced expression of *crtB* and *crtP* (encoding the phytoene synthase and phytoene desaturase, respectively) was reduced in *rppA*-KO compared with the wild-type strain. The *crtB* and *crtP* genes are key for CARs biosynthesis, and their reduced expression can explain the lower CARs content in the *rppA*-KO under moderate light intensity. The effect of RppA on *crtB* and *crtP* expression was due to the direct interaction of the transcription factor with their promoter regions. RppA regulation of *crtB* and *crtP*, as well as *chlG*, was also observed in *Synechococcus* sp. PCC 7002 (a marine cyanobacterium), supporting a conserved functionality of RppA in regulating Chl and CAR biosynthesis in cyanobacteria.

In conclusion, Yu et al. (2024) characterized RppA as a transcription factor regulating the level of photosynthetic pigments under fluctuating light conditions in cyanobacteria (Fig.). Using different techniques, they confirmed RppA binding to the *chlG*, *crtB*, and *crtP* promoters, which encode for key metabolic enzymes in the biosynthesis of Chl and CARs, respectively. This transcriptional control balances the trade-off between photosynthesis and photoprotection, directly impacting cyanobacteria survival and growth under fluctuating light environments. Overall, Yu and colleagues advance our understanding of the different acclimation mechanisms shaping cyanobacteria ecological niche distribution in nature, with a potential impact on their utilization as biotechnological platforms.

**Figure.**
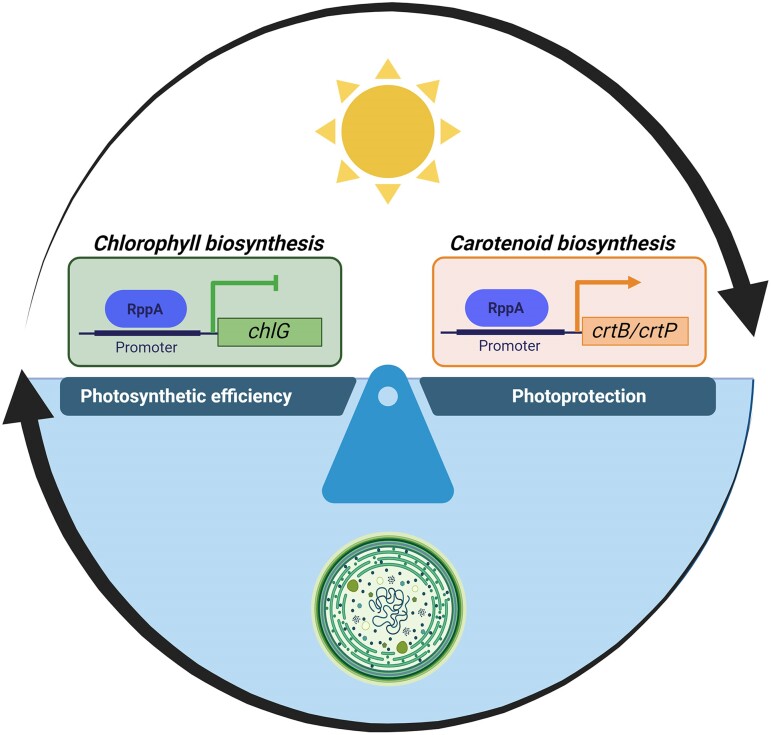
The transcription factor RppA regulates photosynthetic pigment levels in cyanobacteria. RppA binds to the promoter region of *chlG*, repressing chlorophyll biosynthesis, and to the *crtB* and *crtP* promoters, inducing carotenoid biosynthesis. This transcriptional regulation balances the trade-off between photosynthetic efficiency and photoprotection, being fundamental for the acclimation of cyanobacteria to fluctuating light environments. Created in Biorender.

## References

[kiae553-B1] Chidgey JW , Linhartová M, Komenda J, Jackson PJ, Dickman MJ, Canniffe DP, Koník P, Pilný J, Hunter CN, Sobotka R. A cyanobacterial chlorophyll synthase-HliD complex associates with the Ycf39 protein and the YidC/Alb3 insertase. Plant Cell. 2014:26(3):1267–1279. 10.1105/tpc.114.124495 24681617 PMC4001383

[kiae553-B2] Ishii A , Hihara Y. An AbrB-like transcriptional regulator, Sll0822, is essential for the activation of nitrogen-regulated genes in Synechocystis sp. PCC 6803. Plant Physiol. 2008:148(1):660–670. 10.1104/pp.108.123505 18667724 PMC2528100

[kiae553-B3] Knoppová J , Sobotka R, Tichý M, Yu J, Konik P, Halada P, Nixon PJ, Komenda J. Discovery of a chlorophyll binding protein complex involved in the early steps of photosystem II assembly in Synechocystis. Plant Cell. 2014:26(3):1200–1212. 10.1105/tpc.114.123919 24681620 PMC4001378

[kiae553-B4] Kobayashi K , Masuda T. Transcriptional regulation of tetrapyrrole biosynthesis in Arabidopsis thaliana. Front Plant Sci. 2016:7:1811. 10.3389/fpls.2016.01811 27990150 PMC5130987

[kiae553-B5] Li H , Sherman LA. A redox-responsive regulator of photosynthesis gene expression in the cyanobacterium Synechocystis sp. strain PCC 6803. J Bacteriol. 2000:182(15):4268–4277. 10.1128/JB.182.15.4268-4277.2000 10894737 PMC101939

[kiae553-B6] Shalygo N , Czarnecki O, Peter E, Grimm B. Expression of chlorophyll synthase is also involved in feedback-control of chlorophyll biosynthesis. Plant Mol Biol. 2009:71(4–5):425–436. 10.1007/s11103-009-9532-8 19680747

[kiae553-B7] Yu C , Xu H-F, Liu Y-R, Yan W-W, Kong X-L, Zhang Z-C, Dai G-Z, Qiu B-S. The transcription factor RppA 1 regulates chlorophyll and carotenoid biosynthesis to improve photoprotection in cyanobacteria. Plant Physiol. 2024. 10.1093/plphys/kiae502 39321190

[kiae553-B8] Zhi T , Zhou Z, Qiu B, Zhu Q, Xiong X, Ren C. Loss of fumarylacetoacetate hydrolase causes light-dependent increases in protochlorophyllide and cell death in Arabidopsis. The Plant Journal. 2019:98(4):622–638. 10.1111/tpj.14235 30666736

[kiae553-B9] Zhu Y , Graham JE, Ludwig M, Xiong W, Alvey RM, Shen G, Bryant DA. Roles of xanthophyll carotenoids in protection against photoinhibition and oxidative stress in the cyanobacterium Synechococcus sp. strain PCC 7002. Arch Biochem Biophys. 2010:504(1):86–89. 10.1016/j.abb.2010.07.007 20638360

